# Evolutionary Rate Variation among Lineages in Gene Trees has a Negative Impact on Species-Tree Inference

**DOI:** 10.1093/sysbio/syab051

**Published:** 2021-07-13

**Authors:** Mezzalina Vankan, Simon Y W Ho, David A Duchêne

**Affiliations:** 1 School of Life and Environmental Sciences, University of Sydney, NSW 2006, Australia; 2 Research School of Biology, Australian National University, ACT 2601, Australia; 3 Centre for Evolutionary Hologenomics, University of Copenhagen, Copenhagen 1352, Denmark

## Abstract

Phylogenetic analyses of genomic data provide a powerful means of reconstructing the evolutionary relationships among organisms, yet such analyses are often hindered by conflicting phylogenetic signals among loci. Identifying the signals that are most influential to species-tree estimation can help to inform the choice of data for phylogenomic analysis. We investigated this in an analysis of 30 phylogenomic data sets. For each data set, we examined the association between several branch-length characteristics of gene trees and the distance between these gene trees and the corresponding species trees. We found that the distance of each gene tree to the species tree inferred from the full data set was positively associated with variation in root-to-tip distances and negatively associated with mean branch support. However, no such associations were found for gene-tree length, a measure of the overall substitution rate at each locus. We further explored the usefulness of the best-performing branch-based characteristics for selecting loci for phylogenomic analyses. We found that loci that yield gene trees with high variation in root-to-tip distances have a disproportionately distant signal of tree topology compared with the complete data sets. These results suggest that rate variation across lineages should be taken into consideration when exploring and even selecting loci for phylogenomic analysis.[Branch support; data filtering; nucleotide substitution model; phylogenomics; substitution rate; summary coalescent methods.]

Phylogenetic analyses of molecular sequence data have been instrumental in resolving evolutionary relationships across the tree of life and are now benefiting from the growing availability of genome-scale data sets. Phylogenomic inference is often carried out using methods that infer the species tree based on sequence data from a large set of loci. These loci might individually support gene trees that differ from each other and from the underlying species tree (Jeffroy et al. 2006). Any incongruence among gene trees can be treated as the outcome of incomplete lineage sorting under the multispecies coalescent (Maddison 1997; Degnan and Rosenberg 2009), gene flow between lineages (Leaché et al. 2014; Cai et al. 2020), gene duplication (Morel et al. 2020), recombination (Lanier and Knowles 2012), or estimation error (Gatesy and Springer 2014). The practice of minimizing gene-tree incongruence by careful data curation and model selection, while maintaining computational tractability, has been a longstanding matter of interest in phylogenetics (Philippe et al. 2011; Bravo et al. 2019).

To address the problem of computational intractability when dealing with phylogenomic analyses of large data sets, some researchers have suggested using a “data filtering” or “gene shopping” approach (Chen et al. 2015b; Doyle et al. 2015). This method involves selecting a subset of the data that is still likely to generate an accurate estimate of the phylogeny, thereby reducing computational demand while still allowing complex evolutionary models to be used for analysis (Molloy and Warnow 2018). For example, loci might be selected according to their information content and phylogenetic signal. These properties of the sequence data are influenced by a number of factors, including the rate at which the sequences have evolved and the timescale of the process (Duchêne et al. 2018b; Xia et al. 2003; Townsend and Leuenberger 2011; Klopfstein et al. 2017; Steel and Leuenberger 2017). However, the overall substitution rate of a locus does not necessarily show a clear relationship with the accuracy of the inferred tree topology (Aguileta et al. 2008). This is because the phylogenetic signal at any given locus can be obscured by various forms of heterogeneity, such as variation in rates across sites and across lineages (Su and Townsend 2015; Dornburg et al. 2019). Furthermore, estimates of substitution rates at individual loci can be misled by a number of methodological factors, including errors in the model specification (Sullivan and Joyce 2005), alignment, orthology assignment, or sequencing (Wilkinson 1996; Sanderson and Shaffer 2002).

Previous studies of the phylogenetic signal across loci focused on differences in their substitution rates (Yang 1998; Townsend et al. 2012; Klopfstein et al. 2017), but rate variation across lineages can also affect the topological signal (Dornburg et al. 2019). For example, gene trees with high rate variation across lineages tend to have a greater percentage of nodes that conflict with the species tree than do gene trees with low rate variation across lineages (Doyle et al. 2015). In addition, any differences in evolutionary rates across loci and among lineages will ultimately be reflected in the estimates of branch lengths, which are closely linked to the estimate of tree topology. For example, long branches can have negative impacts on phylogenetic accuracy because of their tendency to be grouped together (“long-branch attraction”; Anderson and Swofford 2004). Even a single long branch can drastically change the phylogenetic signal in the data (Su and Townsend 2015). On the other hand, the presence of short branches due to low substitution rates can lead to large amounts of phylogenetic estimation error (Yang 1998). The extent to which rate variation across lineages affects topological signal has been characterized in only a few specific cases (e.g., Doyle et al. 2015; Kuang et al. 2018), but not across a broad range of phylogenomic data sets.

An alternative predictor of phylogenetic accuracy is the ratio of the lengths of internal branches to terminal branches, also known as stemminess (Fiala and Sokal 1985). Low stemminess has previously been associated with a poor topological signal (e.g., Penny et al. 2001; Duchêne et al. 2018c), yet it is frequently observed in phylogenetic trees (e.g., Phillimore and Price 2008). Some explanations for low stemminess include rapid diversification events (McPeek 2008), sparse taxon sampling (Penny et al. 2001; Cusimano and Renner 2010), underparameterization of the substitution model (Revell et al. 2005), and model misspecification due to recombination (Maddison 1997; Degnan and Rosenberg 2009). Despite stemminess being common in empirical data, the extent to which it affects estimates of tree topology in genome-scale data remains unclear.

Testing the link between characteristics of branch lengths and estimates of tree topology across loci has potential benefits for the design of phylogenomic studies. This is likely to be true for data-filtering methods, where the phylogenetic signal from individual loci has a greater impact on species-tree inference. Some of the criteria that have been used for data filtering include phylogenetic branch supports (Blom et al. 2017), the amount of missing data (Molloy and Warnow 2018), measures of substitution model adequacy (Duchêne et al. 2018c; Richards et al. 2018), and base composition (Dávalos and Perkins 2008; Martijn et al. 2018). It is not clear which of these criteria is the most effective (Molloy and Warnow 2018), but it is likely that no single criterion is universally applicable (Reddy et al. 2017); some criteria might even promote the selection of loci that mislead phylogenetic inference (Brown and Thomson 2018; Duchêne et al. 2018c). Nonetheless, branch lengths provide an estimate of the amount of genetic change that is captured in a data set, so it is reasonable to surmise that they have some association with the accuracy of estimates of tree topology (Klopfstein et al. 2017).

In this study, we explore the association between three branch-length metrics and mean branch support estimated for each locus with the inferences of species trees based on a summary-coalescent method. We examine a collection of 30 phylogenomic data sets that represent a range of taxa and genomic data types. We find that gene trees with high among-lineage rate variation are, on average, more dissimilar to other gene trees and to species trees inferred using complete data sets. Our results show that phylogenomic studies are likely to benefit from considering among-lineage rate heterogeneity in gene trees, particularly when the goal is to examine the impact of excluding loci with the poorest signals for inferring species trees.

## Materials and Methods

### Phylogenomic Analyses

We collected a set of 30 phylogenomic data sets covering a wide range of taxa and data types (Table 1), including intron and exon regions, ultraconserved elements, and anchor-enriched regions. The original studies varied widely in their treatment of these data sets. For instance, some studies considered the trees from each of the codon positions of protein-coding loci independently. We followed the data treatments used in the original studies so that our analyses would reflect the approaches that have been used in practice.

**Table 1.** Phylogenomic data sets for which the association between phylogenetic signal and branch characteristics was tested

Taxon	Original number of loci	Final number of loci	Original number of taxa per locus	Final number of taxa per locus	Data type/genomic region	Codon position	Source
Stinging wasps (Aculeata)	807	390	183	21	UCE		Branstetter et al. (2017)
Metazoa	424	260	75	27	Exon	1, 2, 3	Cannon et al. (2016)
Laurasiatherian mammals (Laurasiatheria)	10,259	6298	23	14	Intron	1,2, 3	Chen et al. (2017)
Laurasiatherian mammals (Laurasiatheria)	3638	1386	23	12	CDS		Chen et al. (2017)
Amniote vertebrates (Amniota)	1145	1145	10	10	UCE		Crawford et al. (2012)
Marsupial mammals (Marsupialia)	1535	1093	45	40	Exon	1, 2, 3	Gatesy and Springer (2014)
Butterflies (Papilionoidea)	352	91	205	105	Exon	1, 2, 3	Hughes et al. (2018)
Ray-finned fishes (Actinopterygii)	491	369	27	7	UCE		Irisarri et al. (2018)
North American tarantulas (*Aphonopelma*)	581	310	83	44	Anchor		Duchêne et al. (2018c)
Spiders (Araneae)	327	159	34	22	Anchor		Duchêne et al. (2018c)
North American mygalomorph spiders (Euctenizidae)	403	260	25	19	Anchor		Duchêne et al. (2018c)
Ray-finned fishes (Actinopterygii)	1105	698	298	55	Exon	1, 2,3	Hughes et al. (2018)
Cichlid fishes (Cichlidae)	533	298	149	125	Anchor		Irisarri et al. (2018)
Birds (Aves)	8293	5544	52	24	Exon	1, 2	Felsenstein (1981)
Birds (Aves)	8287	5379	52	25	Exon	3	Felsenstein (1981)
Birds (Aves)	2515	1279	52	23	Intron		Felsenstein (1981)
Gobioid fishes (Actinopterygii: Gobioidei)	570	570	43	43	Exon	1, 2,3	Kuang et al. (2018)
Iguanas (Phrynosomatidae)	583	471	11	11	UCE		Leaché et al. (2015)
Flowering plants (Angiospermae)	461	361	35	35	Anchor		Léveillé-Bourret et al. (2018)
Mosses (Bryophyta)	105	57	146	78	Exon	1, 2, 3	Liu et al. (2019)
Birds (Neoaves)	1541	558	33	27	UCE		McCormack et al. (2013)
Songbirds (Passeri)	515	515	106	106	UCE		Moyle et al. (2016)
Acorn ants (*Temnothorax*)	2098	963	50	30	UCE		Prebus (2017)
Birds (Aves)	259	204	200	191	Anchor		Prum et al. (2015)
Gymnosperms (Gymnospermae)	1308	1308	38	38	Exon	1	Ran et al. (2018)
Gymnosperms (Gymnospermae)	1308	1308	38	38	Exon	2	Ran et al. (2018)
Gymnosperms (Gymnospermae)	1308	1308	38	38	Exon	3	Ran et al. (2018)
Harvestmen spiders (Ischiropsalidoidea)	672	671	5	5	Exon	1	Richart et al. (2016)
Harvestmen spiders (Ischiropsalidoidea)	672	671	5	5	Exon	2	Richart et al. (2016)
Harvestmen spiders (Ischiropsalidoidea)	672	671	5	5	Exon	3	Richart et al. (2016)

*Notes*: The treatment of data sets was similar to that in the original studies.

For each phylogenomic data set, we selected a subset of loci that maximized the product of the number of taxa and the number of loci, while maintaining full occupancy of the data matrix (for details on this procedure, see github.com/mezzalinapaige/rtt_topo). We then inferred the phylogeny for each locus (i.e., the gene tree) with the GTR$+\Gamma $ substitution model using IQ-TREE version 1.6.12 (Nguyen et al. 2015).

**Figure 1.**
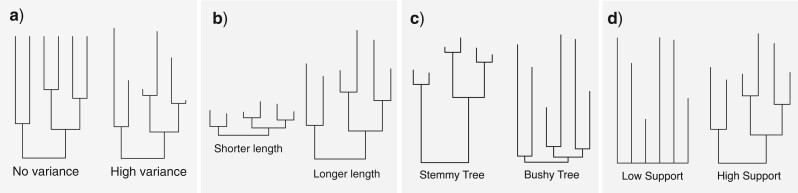
Four branch statistics used to test branch-length signal in each gene tree: a) coefficient of variation in root-to-tip distances, which provides a measure of rate variation across lineages, or inaccuracies in branch-length estimation; b) total tree length (calculated as the sum of branch-lengths), which indicates the overall substitution rate at a locus; c) stemminess, defined as the ratio of internal to terminal branch lengths; and d) Shimodaira—Hasegawa-like approximate likelihood-ratio test (SH-aLRT) of mean branch supports, which can be taken as a measure of the consistency of the topology signal across alignment sites.

We calculated three test statistics that describe the branch-length signal in each gene tree (Fig. 1): 1) the coefficient of variation (CoV) in distances from the midpoint-root to the tips, which provides a measure of rate heterogeneity across lineages; 2) tree length calculated as the sum of all branch lengths; and 3) tree stemminess, the ratio of internal to terminal branch lengths (Degnan and Rosenberg 2009). In addition, we calculated for each gene tree the mean of the statistical support across branches, using the Shimodaira-Hasegawa-like approximate likelihood-ratio test (SH-aLRT; described in Anisimova and Gascuel 2006) using IQ-TREE. This metric provides information about the consistency in the signal of a given branch across the sites in the locus. High values indicate that there is a concordant signal across a large number of the informative sites. Low values occur in loci that have few informative sites or high degrees of rate heterogeneity across sites, or that are affected by saturation or intragenic recombination. We also calculated the number of variable sites for each locus but did not retain this variable in further analyses because it has a strong association with the SH-aLRT mean branch support metric.

We assessed whether the four branch statistics could explain two different measures of whether the inferred gene trees shared the topological signal of the species tree. The first measure was the topological distance from each gene tree to a “reference” species tree that was estimated from the complete data set from the corresponding study, using a summary-coalescent analysis in ASTRAL-III (Zhang et al. 2018). This topological distance quantifies the concordance between the phylogenetic signal in each gene tree and the signal of species history as taken from the complete data set. The second measure of distance from the overall data set was the mean topological distance between the gene tree and each of the other gene trees from the corresponding data set. This evaluates the concordance of the signal in each gene tree with the remainder of the phylogenetic signals across the genome. All topological distances were calculated using the normalized Robinson-Foulds metric (Robinson and Foulds 1981; Penny and Hendy 1985).

We used multiple linear regression to test whether the two measures of distance to the overall signal are explained by each of the four branch statistics. For each of the two response variables (topological distance of the gene tree to the species tree and mean topological distance to other gene trees), we tested a model that included the full set of loci from across the 30 data sets ($N = 34$,662).

Since we aimed to identify the correlates of phylogenetic signal within each data set, we considered the differences in the results and sample sizes across data sets. We included a random factor in each regression model that indicated the source study of each locus, allowing us to account for differences in patterns that might occur among data sets. In this model including all data sets, we corrected tree length for the number of taxa by dividing it by the number of branches in the tree (to obtain the mean of branch lengths) so that the values fell on a similar scale across studies. We also explored the model when weighting each locus by the number of taxa in its source data set, such that data sets with a greater number of loci have a greater contribution to the model.

To focus further on the results for each data set, we performed a second set of regression models where each of the phylogenomic data sets was examined independently. For each data set, we tested whether our two response variables (distances to the overall signal in the data) were explained by our four branch statistics. Therefore, this second set of analyses included two regression tests for each of the 30 data sets that we examined. In these regression models, tree length was left uncorrected for the number of branches.

### Impacts of Data Filtering

To evaluate the practical implications of the branch statistics identified as having the most dominant impact in our regression model on species-tree inference, we inferred the species tree using subsets of the data chosen according to these metrics. For each of the 30 phylogenomic data sets, we selected subsets representing 20%, 40%, 60%, and 80% of the loci. The significant branch metrics were CoV in root-to-tip distances and SH-aLRT mean branch support. A third metric was included as a type of control for verifying that the data behaved as expected: the normalized Robinson-Foulds distance from each gene tree (locus) to its respective species tree. For each of the three metrics, we selected loci starting from the “top” locus (descending rank order), then we selected loci starting from the “bottom” locus (ascending rank order). We also selected subsets of the data by sampling loci randomly. This procedure produced 28 subsets of loci from each of the 30 phylogenomic data sets.

We used a summary-coalescent approach in ASTRAL-III to estimate the species tree from each of the subsets of the data. The species-tree estimates from these data subsets were then compared with the overall signal in the data set, using their normalized Robinson-Foulds distance from the species tree inferred from the full data set, or “reference” tree. As a secondary measure of performance, we considered the mean branch supports of the species-tree estimates from the data subsets compared with that of the reference species tree.

We tested for differences in the signals of the species-tree inferences from the top-ranked, bottom-ranked, and randomly selected loci. We performed this analysis in R using a one-way ANOVA and further evaluated the differences between methods of data filtering using a Tukey HSD post-hoc multiple-comparison test.

**Figure 2.**
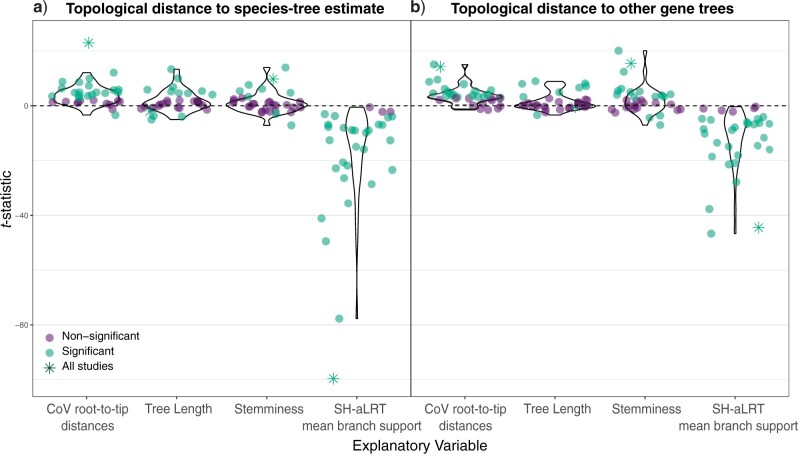
Summary $t$-statistic for multiple-regression tests of the association between four explanatory variables describing branches and each of two response variables: a) topological distance between gene trees and the inferred species tree; and b) mean distance from each gene tree to all other gene trees. Circle markers represent the $t$-statistics derived from regression analyses of individual data sets. Star markers indicate the results of the analyses that include all 30 phylogenomic data sets. Green markers indicate a significant association ($P < 0.05$) between the branch statistic and the topological distance for the given data set, while purple markers represent data sets with no such association. For visualization, markers have been jittered horizontally.

## Results

### Phylogenomic Analyses

The regression analyses that included the full set of loci from 30 phylogenomic data sets showed that some of our explanatory variables (branch-based characteristics) had a significant association with both measures of distance to the overall signal in the data (topological distance to the species tree and topological distance to other gene trees; Fig. 2). Specifically, we found that both metrics of distance to the overall signal have a positive association with the CoV in root-to-tip distances, and a negative association with mean SH-aLRT branch support. The association was strongest between the two metrics of distance to the overall signal and mean SH-aLRT branch support. Strikingly, we found limited evidence for an association between distance to the overall signal and tree length or stemminess. Results were similar across regression models in which samples (loci) were weighted by number of branches or by number of taxa in their respective data sets (Supplementary Fig. S1 available on Dryad at http://dx.doi.org/10.5061/dryad.c866t1g61).

**Figure 3.**
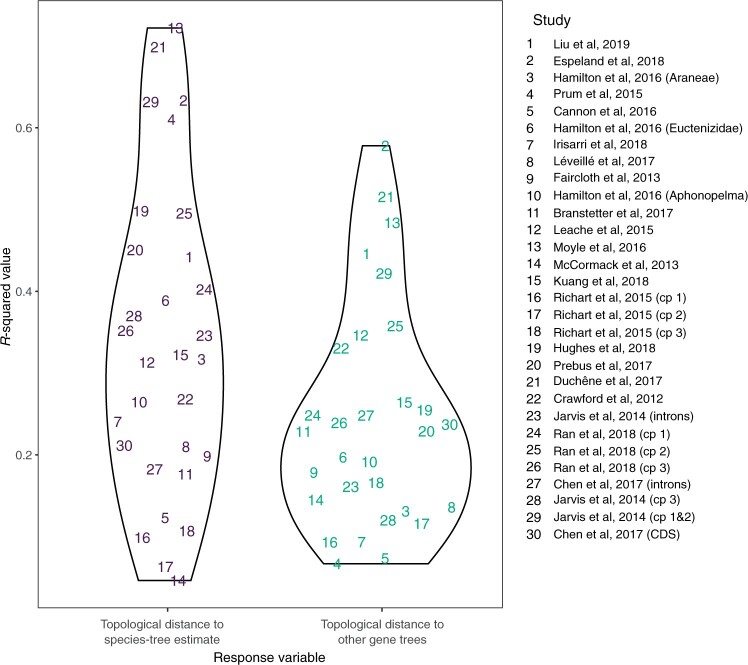
$R^{2}$ values for each of the two multiple-regression tests of the association between the four branch statistics and our two response variables: a) topological distance between gene trees and the inferred species tree; and b) mean distance from each gene tree to all other gene trees. Each number denotes a phylogenomic data set to which pairs the regression model was applied. For visualization, markers have been jittered horizontally.

The regression models that explored individual data sets supported the results from our larger regression models. Only a small minority of data sets showed an effect opposite to those observed for the CoV in root-to-tip distances and branch support. Meanwhile, there was substantial variation in terms of the association between the distance to the overall signal in the data set and tree length or stemminess. The $t$-statistics were similar among regression models with each of the two measures of distance to the overall species-tree signal in the data (Supplementary Fig. S2 available on Dryad). $R$-squared values for each of the regression models varied widely among the data sets but were frequently $>$0.5 (Fig. 3). The direction and strength of effect sizes were similar between the two measures of distance to the overall species-tree signal in the data.

### Impacts of Data Filtering

**Figure 4.**
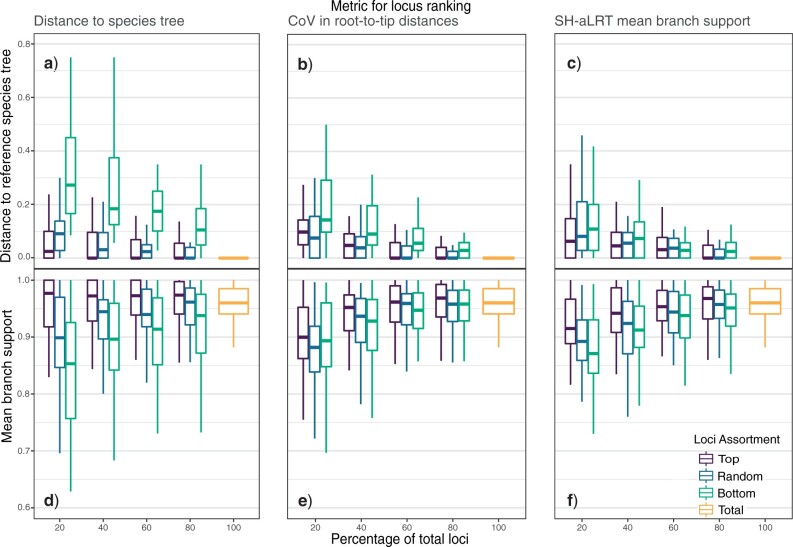
Performance of species-tree inference from loci from each of the 30 phylogenomic data sets, from subsets of various sizes of each original data set (x-axis). Loci filtered according to three properties of their gene trees: a, d) distance to the reference tree; b, e) CoV in root-to-tip distances; and c, f) mean branch support. Each series of boxplots shows results from the top-ranked loci (purple), bottom-ranked loci (green), and a random sample of loci of each size (blue) for each of the 30 phylogenomic data sets. a—c) The distance of each species tree inferred from data subsets to the reference species tree. d—f) The mean branch support in species trees inferred from each data subset.

The species trees inferred from subsets of each of the 30 data sets, based on loci ranked by either ascending or descending values of CoV in root-to-tip distances and SH-aLRT mean branch support, showed similar patterns across all types of rankings (Fig. 4). Species trees estimated using the top-ranked loci resulted in topologies that were more similar to those of the reference species trees and had higher mean branch supports than trees estimated from a random sample of loci. Analyses of the bottom-ranked loci produced species-tree estimates that were considerably more distant to the reference trees, with lower mean branch supports, when compared with those estimated from top-ranked or randomly selected loci. In addition, species trees inferred from the bottom-ranked loci showed greater variance in both metrics of distance to the overall signal than those inferred from other subsets of loci. Species trees inferred from the top-ranked loci generally had less variation than those inferred from a random sample of loci. While these patterns were consistent across the different percentages of loci, the subsets with the highest numbers of loci (i.e., 60% and 80%) produced estimates of the species tree with the smallest distances to the reference species tree. We found that these trees had higher mean branch supports than the reference tree and were often topologically identical to the reference tree.

We also found that the results from filtering different percentages of the data were significantly different (Supplementary Table S2 available on Dryad). For loci ranked by their CoV in root-to-tip distances and SH-aLRT mean branch support, distinctions between locus rankings (i.e., top-ranked, bottom-ranked, and randomly chosen loci) were strongly associated with distance to the reference tree. These results were significant across all the different percentages of locus subsets (20%, 40%, 60%, and 80%). However, there was only a significant change in the mean branch support of the species tree when 20% or 40% of loci were used.

The results of our post-hoc multiple-comparisons test revealed that only a small number of pairs of methods of filtering data were significantly different. Loci with the highest CoV in root-to-tip distances produced species-tree inferences with significantly greater distance to the reference tree when compared with trees estimated using randomly chosen loci and loci with low CoV in root-to-tip distances (Supplementary Table S3 available on Dryad). The resulting species trees had smaller topological distances to the reference tree but, strikingly, had similar mean branch supports. This result was consistent across the different percentages of loci with the bottom-ranked CoV in root-to-tip distances, even as the number of better-ranked loci increased. Indeed, we found no significant difference between the species trees inferred from the loci with the top-ranked CoV in root-to-tip distances and those inferred using a random sample of loci, nor any differences when loci were ranked according to SH-aLRT mean branch support.

The results of the statistical tests for data subsets ranked by CoV in root-to-tip distances aligned with those observed from our control ranking, using distance of gene trees to the reference species tree. We found that species trees estimated from the bottom-ranked loci were significantly more distant from the reference tree, whilst analyses of the top-ranked loci produced species trees with no statistical difference from those inferred from a random sample of loci. This pattern was consistent across the different sizes of data subsets. The main difference between the species trees inferred from the control data and our branch statistics was a significant change in our other tested metric, mean branch support. For subsets with 20%, 40%, and 60% of bottom-ranked loci, the mean branch support of species trees was lower than those for species trees estimated using the top-ranked loci and all (“total”) loci. The only statistical difference in mean branch support when loci were ranked according to distance to the reference tree occurred in subsets of the “top” 20% of loci. However, these inferred species trees showed no change in the topological distance to the reference tree.

## Discussion

Our analysis of a diverse collection of phylogenomic data sets shows that gene trees with high variation in root-to-tip distances and low mean branch supports are associated with greater distance to the species-tree topology inferred from each data set as a whole. Strikingly, gene-tree length is a poor predictor of the overall species-tree signal in a data set. This is surprising because tree length is proportional to the overall substitution rate at a locus (Yang 1998) and is a prominent form of variation in the phylogenetic information across gene trees (Duchêne et al. 2020). However, our results are consistent with recent work that has emphasized the importance of heterogeneity in the data rather than the overall substitution rate as an indicator of phylogenetic accuracy (Su and Townsend 2015; Dornburg et al. 2019). Our analyses also suggest that removing a small percentage of loci with high variation in root-to-tip distances can result in species-tree inferences that are more similar to those estimated using a complete data set of loci, than when analyzing random subsets of loci. These loci are filtered according to the signal of branch lengths rather than tree topology. While inferring the “true” species tree can still pose a challenge for any filtered data set, finding an objective method to extract the dominant signal in the data is often a primary aim in phylogenetic analyses. Such an interpretation of data filtering also relies on gene-tree estimates that accurately represent independent gene histories and follow the multispecies coalescent (Mendes et al. 2019). Our analyses further demonstrate that the choice of criterion for data filtering is likely to be critical when small subsets of genomic data are used, but that this choice is less important when large numbers of loci are included.

The performance of species-tree inference can potentially be improved by removing loci with particular patterns of rate variation across lineages (Kuang et al. 2018). High variation in root-to-tip distances in gene trees might be the result of analyzing loci with complex or poorly modeled signals. The removal of these signals from a data set has the potential to reduce the stochastic error associated with the topology and thereby improve species-tree estimation (Jeffroy et al. 2006; Doyle et al. 2015; Brown and Thomson 2017). A formal method of identifying loci with constant rates across lineages is to compare a model of rate constancy versus one allowing rate variation (Felsenstein 1981), also known as a likelihood-ratio test of clocklikeness. To allow for acceptable levels of among-lineage rate variation, one approach that might benefit phylogenomic studies is to use a more stringent threshold for rejecting clocklikeness (e.g., Felsenstein’s likelihood-ratio test with $\alpha = 0.0001$), such that researchers can explore their phylogenomic data by excluding only the loci that have extreme amounts of among-lineage rate heterogeneity.

We found that gene-tree branch supports have a strong association with the topology of the inferred species tree, but filtering loci on this basis does not strengthen the signal of the species tree inferred from the full set of loci. Because SH-aLRT mean branch support is a measure of the consistency of a signal for a given branch across sites, this result suggests that disagreement among sites has a limited influence on species-tree inference compared with rate heterogeneity across lineages and sites (Dornburg et al. 2019). Gene-tree error arising from disagreement within gene regions becomes less important as the underlying signal of the species tree emerges with increasing number of loci. The lack of a stronger signal of the reference species tree in loci filtered according to SH-aLRT may be due to dominant signals within loci driving the estimate of the topology. Previous work has shown that gene trees with high bootstrap branch supports are associated with greater nodal support values in inferred species trees (Blom et al. 2017). Nonetheless, there are substantial differences among metrics of branch support, and they are likely to differ in their performance. Although we have focused here on mean SH-aLRT branch support, detailed evaluations of other branch-support metrics would be beneficial for identifying optimal strategies for data filtering for phylogenomics (e.g., Lemoine et al. 2018; Minh et al. 2020).

The results of this study are likely to have been affected by the choice of models used for analyses. For example, variation in root-to-tip distances that leads to poor phylogenetic accuracy might be due to poor substitution model performance rather than biological sources of rate variation among lineages. Sequence evolution might violate the assumptions of the most commonly used models, for example, due to heterogeneity in the evolutionary process that leads to large differences in base composition across taxa (Jermiin et al. 2004; Doyle et al. 2015; Martijn et al. 2018). Variation in root-to-tip distances can also be symptomatic of factors causing inaccurate estimates of branch lengths. Sequences that have evolved under a strict molecular clock are expected to yield gene trees with uniform root-to-tip distances (i.e., an ultrametric tree). However, trees are unlikely to be inferred as ultrametric when branch lengths are estimated poorly. Some of these potential problems can be detected by using tests of model adequacy (Brown and ElDabaje 2009; Doyle et al. 2015; Duchêne et al. 2020; Duchêne et al. 2018b,c).

Inference of species trees in this study was done using a summary-coalescent method that benefits from using large numbers of loci (Streicher et al. 2015). Other methods of phylogenomic inference might respond differently to the size of the data set, with superior performance in analyses of small numbers of taxa (e.g., locus concatenation; Streicher et al. 2015) or large numbers of individuals per species (Ogilvie et al. 2017; Cao et al. 2019). Therefore, when selecting subsets of data for phylogenomic inference, researchers should carefully consider the methods and models that are to be used (Bravo et al. 2019).

Poor phylogenetic inferences in the presence of high variation in root-to-tip distances or low branch supports might also be an artifact of data preparation rather than poor model performance. If model performance were a primary driver of our observed cases of low similarity between gene trees and inferred species trees, then we would expect poor accuracy to be strongly associated with low stemminess (Revell et al. 2005). One way to mitigate errors in data preparation is to identify and remove any taxa that have highly variable positions across gene trees (“rogue taxa”; Aberer et al. 2013) or that sit on extremely long terminal branches (Mai and Mirarab 2018). Similarly, phylogenomic studies of the relationships at a specific branch of the tree can benefit from identifying loci with a highly decisive signal (Dornburg et al. 2019) or those with the signal of a long branch separating the taxa in question (Chen et al. 2015a). Given that multiple factors can affect branch-length estimates, any problematic loci or lineages should ideally be identified using a mixture of methods.

The results of our study offer a basis for developing a framework for phylogenomic analysis that prioritizes the removal of loci with a signal of high variation in root-to-tip distances. In the era of whole-genome data sets, these forms of data filtering are likely to be useful when the intention is to employ parameter-rich evolutionary models, such as those used to estimate divergence times and any methods based on the Bayesian framework. Our results suggest that, on a per-locus basis, the difference between a gene tree and the dominant topological signal in genomic data depends more on the adequacy of the evolutionary model and homogeneity of rates among lineages than on the mean substitution rate or conflicting signals among sites. Potential avenues for future research include exploring the importance of model adequacy when estimating branch lengths or comparing the performance of various metrics of branch support for predicting phylogenetic accuracy. Further examination of the correlates of reliable phylogenetic signal will be useful in guiding the selection of loci for phylogenomic analyses.

## Supplementary Material

Data available from the Dryad Digital Repository: http://dx.doi.org/10.5061/dryad.c866t1g61.
